# Association Between Minor Salivary Gland Biopsy During Sjӧgren’s Syndrome and Serologic Biomarkers: A Systematic Review and Meta-Analysis

**DOI:** 10.3389/fimmu.2021.686457

**Published:** 2021-06-11

**Authors:** Onorina Berardicurti, Piero Ruscitti, Paola Di Benedetto, Settimio D’Andrea, Luca Navarini, Annalisa Marino, Paola Cipriani, Roberto Giacomelli

**Affiliations:** ^1^ Department of Biotechnological and Applied Clinical Sciences, Rheumatology Unit, University of L’Aquila, L’Aquila, Italy; ^2^ Department of Life, Health and Environment Sciences, Andrology Unit, University of L’Aquila, L’Aquila, Italy; ^3^ Rheumatology and Immunology Unit, Department of Medicine, University of Rome ‘Campus Biomedico’, Rome, Italy

**Keywords:** Sjӧgren’s syndrome, minor salivary gland biopsy, focus score, germinal center, clinical features, serological biomarkers, autoantibodies

## Abstract

**Objective:**

Patients with primary Sjögren’s syndrome (pSS) may develop a potentially severe disease with extra-glandular involvement and lymphoma insurgence. Minor salivary gland biopsy is routinely used in the disease diagnosis, but its potential role as a biomarker for clinical disease presentation and prognosis is still poorly understood.

**Methods:**

We performed a systematic review and meta-analysis on clinical presentation and prognosis in pSS patients who underwent minor salivary gland biopsy at diagnosis according to the PRISMA guidelines.

**Results:**

We included five retrospective studies and 589 pSS patients. Ectopic GCs presence was not associated with a significant increase in the odds ratio for the clinical variables explored such as salivary gland swelling, arthritis, and Raynaud’s phenomenon. As far as serological features are concerned, ectopic GCs presence accounted for an increased ratio of antibodies anti-SSA (OR = 3.13, 95% CI: 1.25–7.85, p = 0.02, I^2^ = 79%), anti-SSB (OR = 3.94, 95% CI: 1.50–10.37, p = 0.0005, I^2^ = 80%), and RFs presence (OR = 3.12, 95% CI: 1.94–5.00, p < 0.00001, I^2^ = 0%).

**Conclusions:**

This study showed that the association between ectopic GC in salivary glands identifies a clinical subset characterized by autoantibodies presence, and probably pSS patients affected from a more severe disease.

## Introduction

Primary Sjögren’s syndrome (pSS) is an autoimmune disorder characterized by chronic exocrine gland infiltration, sicca syndrome, extra-glandular manifestations, and an increased risk of lymphoma development (1). The disease mainly affects middle-aged women, and its incidence ranges between 3 and 11 per 100,000 individuals per year (1, 2). More than half of the affected patients develop systemic involvement (3, 4). In severe patients, the excess of mortality is mainly related to the development of B cell lymphoma and visceral involvement, like interstitial lung disease, renal failure, hypokalemic paralysis, and severe cryoglobulinemic vasculitis (5). T and B-lymphocytes, together with other immune cells, home into salivary glands, promoting the disease development and determining the specific histologic pattern, known as focal lymphocytic sialadenitis (LFS) (6), used for diagnostic and classificative purposes. Minor salivary glands biopsy (mSGB) represents the main criteria used in the disease classification. In fact, from 2002, when the American–European Consensus Group (AECG) classification criteria for pSS have been proposed, to 2016, when the last set of classification criteria were released, the presence of LFS with a focus score (FS) ≥1 remains the “gold standard” for pSS classification (7–9). The first description of the minor salivary gland infiltrate related to keratoconjunctivitis and sicca syndrome is from H. Sjögren in the 1933 (10). Successively, Chisholm and Mason, Greenspan and Daniels, and Tarpley suggested three different scoring systems for mSGB. Chisholm and Mason proposed a score based on five grades, from 0 to 4, considering the presence of slight or moderate lymphocytic infiltration and/or focus of lymphocytes (11). Greenspan and Daniels introduced in 1974 the concept of LFS and FS. The FS was defined as the number of foci in a 4 mm^2^ area of normal-appearing tissue, and LFS was defined as an FS ≥1 (12). Finally, Tarpley proposed a score including acinar destruction and fibrosis, associated with the number of immune cell aggregates, to graduate mSGB (13). So far, different studies analyzed the role of mSG involvement as a specific tool for diagnosis, and data from a previous systematic literature review showed a wide range of sensitivity and specificity for pSS diagnosis, from 63.5 to 93.7% and from 61.2 to 100%, respectively (14). Furthermore, a large variability was observed concerning the estimated positive predictive value of mSGB, which ranged from 74.2 to 100%, and the estimated predictive negative value, which ranged from 39.1 to 96.1% (14). Despite these wide ranges, the diagnostic role of mSGB in pSS is largely recognized and recommended, while its role in pSS patients’ stratification and systemic disease prognosis still remain poorly known.

On these bases, we designed and conducted a systematic literature review (SLR) and meta-analysis to assess the value of mSGB for pSS patients’ stratification. Furthermore, we explored the possible predictive role of mSGB in systemic disease development.

## Materials and Methods

### Protocol

This study was conducted according to the Cochrane Collaboration and the Preferred Reporting Items for Systematic reviews and Meta-Analyses (PRISMA) statement (15). The PRISMA checklist is presented in **Table 1**.

**Table 1:** PRISMA 2009 Checklist.

Section/topic	#	Checklist item	Reported on page #
**TITLE**			
Title	1	Identify the report as a systematic review, meta-analysis, or both.	1
**ABSTRACT**			
Structured summary	2	Provide a structured summary including, as applicable: background; objectives; data sources; study eligibility criteria, participants, and interventions; study appraisal and synthesis methods; results; limitations; conclusions and implications of key findings; systematic review registration number.	2
**INTRODUCTION**			
Rationale	3	Describe the rationale for the review in the context of what is already known.	4
Objectives	4	Provide an explicit statement of questions being addressed with reference to participants, interventions, comparisons, outcomes, and study design (PICOS).	4
**METHODS**			
Protocol and registration	5	Indicate if a review protocol exists, if and where it can be accessed (*e.g*., Web address), and, if available, provide registration information including registration number.	
Eligibility criteria	6	Specify study characteristics (*e.g.*, PICOS, length of follow-up) and report characteristics (*e.g.*, years considered, language, publication status) used as criteria for eligibility, giving rationale.	4–5
Information sources	7	Describe all information sources (*e.g.*, databases with dates of coverage, contact with study authors to identify additional studies) in the search and date last searched.	5
Search	8	Present full electronic search strategy for at least one database, including any limits used, such that it could be repeated.	5–6
Study selection	9	State the process for selecting studies (*i.e.*, screening, eligibility, included in systematic review, and, if applicable, included in the meta-analysis).	5–6
Data collection process	10	Describe method of data extraction from reports (*e.g.*, piloted forms, independently, in duplicate) and any processes for obtaining and confirming data from investigators.	5–6
Data items	11	List and define all variables for which data were sought (*e.g*., PICOS, funding sources) and any assumptions and simplifications made.	5-6
Risk of bias in individual studies	12	Describe methods used for assessing risk of bias of individual studies (including specification of whether this was done at the study or outcome level), and how this information is to be used in any data synthesis.	5-6
Summary measures	13	State the principal summary measures (*e.g.*, risk ratio, difference in means).	6
Synthesis of results	14	Describe the methods of handling data and combining results of studies, if done, including measures of consistency (*e.g.*, I^2^) for each meta-analysis.	6
Risk of bias across studies	15	Specify any assessment of risk of bias that may affect the cumulative evidence (*e.g.*, publication bias, selective reporting within studies).	6
Additional analyses	16	Describe methods of additional analyses (*e.g.*, sensitivity or subgroup analyses, meta-regression), if done, indicating which were pre-specified.	6
**RESULTS**			
Study selection	17	Give numbers of studies screened, assessed for eligibility, and included in the review, with reasons for exclusions at each stage, ideally with a flow diagram.	6–7
Study characteristics	18	For each study, present characteristics for which data were extracted (*e.g.*, study size, PICOS, follow-up period) and provide the citations.	6–7
Risk of bias within studies	19	Present data on risk of bias of each study and, if available, any outcome level assessment (see Item 12).	6–7
Results of individual studies	20	For all outcomes considered (benefits or harms), present, for each study: (a) simple summary data for each intervention group (b) effect estimates and confidence intervals, ideally with a forest plot.	7
Synthesis of results	21	Present results of each meta-analysis done, including confidence intervals and measures of consistency.	7
Risk of bias across studies	22	Present results of any assessment of risk of bias across studies (see Item 15).	7
Additional analysis	23	Give results of additional analyses, if done (*e.g.*, sensitivity or subgroup analyses, meta-regression [see Item 16]).	7
**DISCUSSION**			
Summary of evidence	24	Summarize the main findings including the strength of evidence for each main outcome; consider their relevance to key groups (*e.g.*, healthcare providers, users, and policy makers).	10
Limitations	25	Discuss limitations at study and outcome level (*e.g.*, risk of bias), and at review-level (e.g., incomplete retrieval of identified research, reporting bias).	11
Conclusions	26	Provide a general interpretation of the results in the context of other evidence, and implications for future research.	10-11
**FUNDING**			
Funding	27	Describe sources of funding for the systematic review and other support (*e.g.*, supply of data); role of funders for the systematic review.	11

From: Moher D, Liberati A, Tetzlaff J, Altman DG, The PRISMA Group (2009). Preferred Reporting Items for Systematic Reviews and Meta-Analyses: The PRISMA statement. PLoS Med 6(7): e1000097. doi:10.1371/journal.pmed1000097.

### Eligibility Criteria

In this study, we included all peer-reviewed published articles that reported demographic, clinical, and serological characteristics of pSS according to their mSGB histology. We selected all the studies conducted in pSS patients with a confirmed diagnosis (Population) who performed a mSGB (Intervention and Control) that reported the demographic, clinical, and serological associated factors (Outcome). We did not introduce temporal limits in our search strategy. Review articles, case reports, opinion articles, letters, brief reports, non-English publications, and those with missing data were excluded.

### Search Strategy and Study Selection

**Figure 1:**
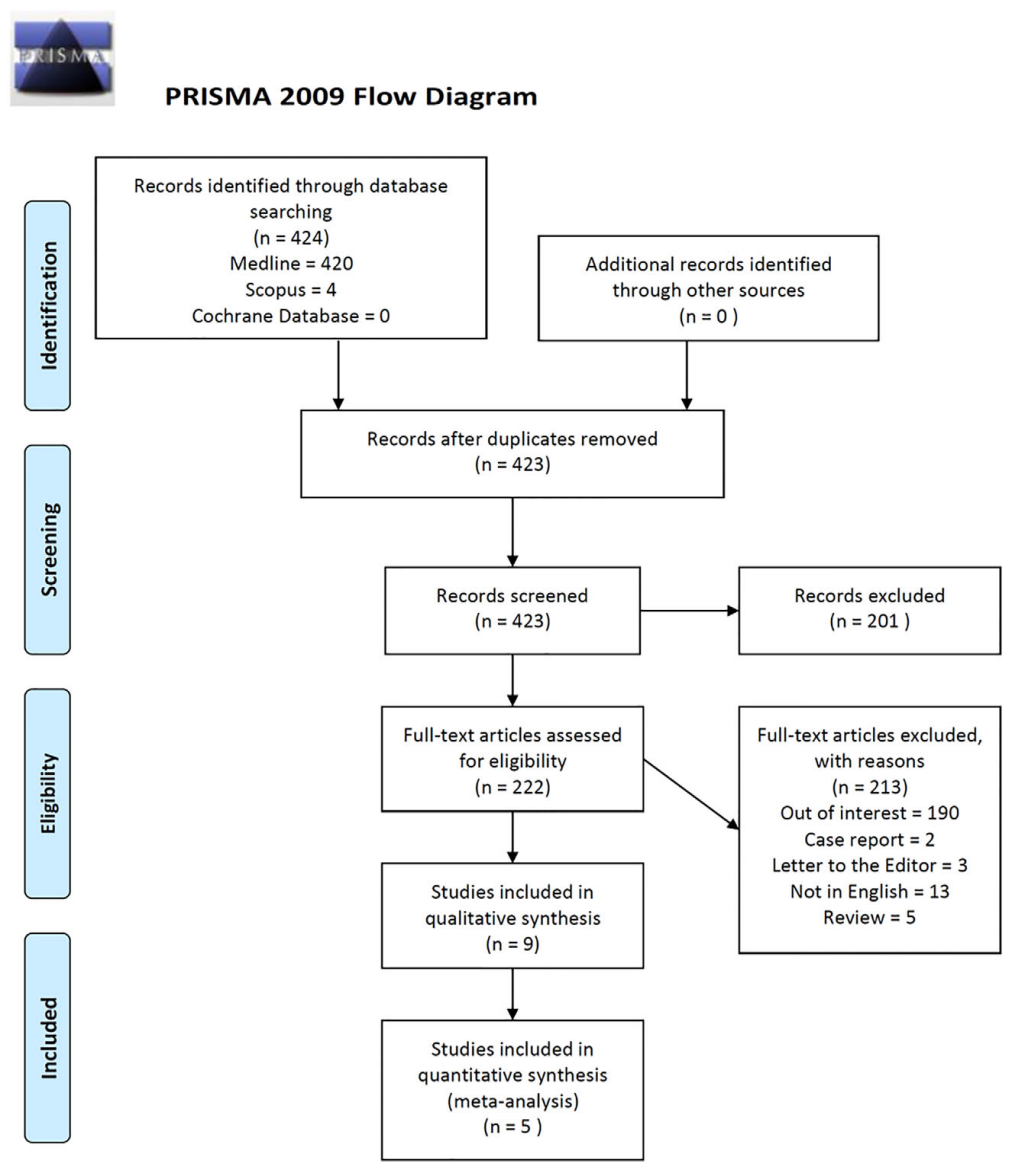
PRISMA 2009 Flow Diagram.

We conducted a systematic search in MEDLINE, Cochrane Library, and SCOPUS databases to identify all relevant English-language publications, with the terms: ((“Histology”[MeSH Terms] AND “Salivary Glands”[MeSH Terms]) OR “focus score” OR “lymphomononuclear infiltrates” OR “Chisholm and Mason” OR “Tarpley”) AND “Sjogren’s syndrome”[MeSH Terms]). Two independent reviewers (OB and PR) first screened the retrieved papers based on the title and abstract (**Figure 1**). If it was not clear from the title and abstract whether the paper contained relevant data, the full paper was retrieved. The list of all excluded papers after full-text assessment is reported in **Supplementary Table 1**. Finally, we scrutinized the reference lists of the identified articles to find additional pertinent studies.

### Data Extraction

Data from the selected articles were extracted according to the first author; publication year; number of participants; number of female; clinical features [xerostomia, xeroftalmia, parotid swelling, arthritis, renal involvement, hematological involvement, lung involvement, cutaneous involvement, peripheral nervous system (PNS) involvement, central nervous system (CNS) involvement, lymphoma, muscular involvement, and Raynaud’s phenomena (RP)], and serological features [anti-SSA antibodies, anti-SSB antibodies, rheumatoid factor (RF), complement components (C3, C4), cryoglobulinemia, hypergammaglobulinemia, leukopenia and anti-nuclear antibodies (ANA)]. Wherever data were missing or inconsistent, the authors were contacted to obtain the necessary information.

### Assessment of Methodological Quality

The quality of studies included in the quantitative analysis was assessed using the “star system” of the Newcastle-Ottawa Quality Assessment Scale (NOS) (16). The minimum and maximum scores that could be awarded were zero stars and nine stars, respectively (**Table 2**). Studies that scored ≥seven stars were regarded as high quality. The quality assessment was performed by two reviewers (OB and PR), and any disagreement was resolved by a third reviewer (SD’A) who re-evaluated the original study.

**Table 2:** Newcastle-Ottawa Assessment Scale.

Study	Selection				Comparability		Exposure			
	***Definition of cases***	***Representativeness of cases***	***Selection of controls***	***Definition of controls***	***On age***	***On otder risk factors***	***Assessment of exposure***	***Same metdods of ascertainment for cases and controls***	***Non -responserate***	**Total score**
Carubbi et al. (17)	★	★	★	☆	☆	★	★	★	☆	6
He et al. (18)	★	★	★	☆	★	★	★	★	☆	7
Jonsson et al. (19)	★	★	★	☆	★	★	★	★	☆	7
Lee et al. (20)	★	★	★	☆	★	★	★	★	☆	7
Rekesten et al. (21)	★	★	★	☆	★	★	★	★	☆	7

★, 1; ☆, 0.

### Statistical Analysis

The relationship between ectopic GCs presence and glandular swelling, arthritis, Raynaud’s phenomena (RP), anti-SSA antibodies, anti-SSB antibodies, or rheumatoid factor (RF) was assessed using odds ratio (OR) and 95% CI as well as Mantel–Haenszel estimates. A significant heterogeneity was expected among studies. Data were combined using random effect models, which assumes that the included studies have varying effect sizes, thus providing a conservative estimate of the overall effect. The Cochrane chi-square (Cochrane Q) test and I^2^ test were carried out to analyze the heterogeneity among the results of different studies. An I^2^ value <25% was considered indicative of no heterogeneity, while I^2^ >50% and/or P <0.05 indicated substantial heterogeneity (17). The extracted data were analyzed using the statistical software R (version 3.0.3; R Foundation for Statistical Computing, Vienna, Austria) and the Review Manager (RevMan) of the Cochrane Library (version 5.3; Copenhagen: The Nordic Cochrane Centre, The Cochrane Collaboration, Copenhagen, Denmark).

## Results

### Study Selection and Characteristics

A total of 424 articles were retrieved by using the above-mentioned search strategy, and after screening titles and abstracts, 222 articles were selected for full-text assessment. After review, nine studies were included in the qualitative and five studies were included in the quantitative analysis. Among the studies included in the quantitative analysis, two studies were conducted in Norway (18, 19); one in Italy (20); one in China (21); and one in Korea (22). All of them referred to pSS patients fulfilling the revised criteria proposed by the American-European Consensus Group (7), and all the studies were retrospective. In all the included research studies the mSGBs were performed and analyzed according to the standard procedures (12). The main characteristics of the selected studies are reported in **Table 3**. The overall quality of the selected studies is high, but all the retrieved studies were retrospectively designed (**Table 2**). The main demographic, clinical, and serologic characteristics of pSS patients are reported in **Tables 3**, **4**.

**Table 3:** Main demographic and clinical characteristics of patients in selected studies.

Focus score																
First Author,Year	Mean Age, Years	N	F	Xerostomia	Xeroftalmia	Glandular swelling	Arthritis	Renal involvement	Hematological involvement	Lung involvement	Cutaneous involvement	PNS involvement	CNS involvement	Lymphoma	Muscular involvement	RP
Wise and Woodruff (23)		187	164													
*FS ≤ 1*	53.6	111	97	67	70	10		6		9	12					
*FS > 1*	56	76	67	47	47	19		5		12	10					
Carubbi et al (24)		383	368													
*FS = 0*		72	69	71	70	28	51	2	25	8	9	7	1	4	0	19
*FS = 1*		74	73	70	60	14	44	3	10	7	10	2	5	2	1	21
*FS > 1*		237	226	212	225	79	150	4	43	16	17	12	3	8	4	61
Daniels et al. (25)		1,726														
*FS ≥ 1*		730		669	624											
*FS < 1*		328		292	292											
Reksten et al. (19)		141														
*FS−*	49	18														
*FS+*	51	97														
*GC*																
Carubbi et al. (20)	52	104	98													
*GC−*	55	46	42			3	19	2		6	5	5	1	0	1	16
*GC+*	49	58	56			19	28	2		6	7	5	4	2	1	18
He et al. (21)		126	124													
*GC−*		90	88			17	40									16
*GC+*	49.92	36	36			8	14									6
Jonsson et al. (18)	54	169														
*GC−*	56	122				32										
*GC+*	52	47				14										
Lee et al. (22)		93	91													
*GC−*	45.08	65	64			5	43	1						0		15
*GC+*	43.39	28	27			3	14	2						1		6
Reksten et al. (19)		97														
*GC-*	53	70														
*GC+*	46	27														

N, numbers of included patients; F, number of female; PNS, peripheral nervous system; CNS, central nervous system; RP, Raynaud’s phneomena.

**Table 4:** Main serological characteristics of patients in selected studies.

Focus score													
First Author,Year	Mean Age, Years	N	F	anti-SSA	anti-SSB	anti-SSA+ anti-SSB	RF	Low C3	Low C4	Cryoglobulinemia	Hypergammaglobulinemia	Leukopenia	ANA
Wise and Woodruff (23)		187	164										
*FS ≤ 1*	53.6	111	97	10	4		24						35
*FS > 1*	56	76	67	35	24		48						52
Carubbi et al. (24)		383	368										
*FS = 0*		72	69	45	1	26	42	8	3		40	26	67
*FS = 1*		74	73	27	1	5	18	9	3		11	13	59
*FS > 1*		237	226	78	5	80	110	38	23		109	77	214
Daniels et al. (25)		1,726											
*FS ≥ 1*		730					458				424		
*FS < 1*		328					64				54		
Reksten et al. (19)		141											
*FS−*	49	18		16	6	4	0						
*FS +*	51	97		40	22	18	14						
*GC*													
Carubbi et al. (20)	52	104	98										
*GC−*	55	46	42	9	2	1	10				6	12	29
*GC+*	49	58	56	49	26	25	23				28	17	51
He et al. (21)		126	124										
*GC−*		90	88	48	12								
*GC+*	49.92	36	36	24	24								
Jonsson et al. (18)	54	169											
*GC−*	56	122		45	24		14	7	11				54
*GC+*	52	47		25	17		10	5	6				22
Lee et al. (22)		93	91										
*GC−*	45.08	65	64	55	25		26						
*GC+*	43.39	28	27	26	12		18						
Reksten et al. (19)		97											
*GC−*	53	70		26	13	11	7						
*GC+*	46	27		14	9	7	7						

N, number of included patients; F, number of female; ANA, anti-nuclear antibodies.

### Germinal Center Influence on Clinical and Serological Features in pSS

**Figure 2:**
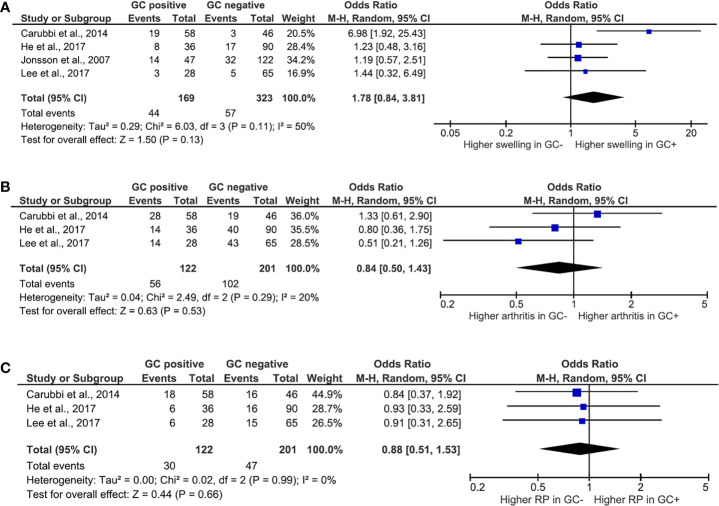
Meta-analysis of the presence of salivary gland swelling **(A)**, arthritis **(B)**, and Raynaud’s phenomena **(C)** between patients with Sjögren’s syndrome with or without ectopic germinal center. The size of squares is proportional to the weight of each study. Horizontal lines indicate the 95% CI of each study; diamond, the pooled estimate with 95% CI; N, the number of persons at baseline; and OR, odds ratio; GC, germinal center; RP, Raynaud’s phenomena.

**Figure 3:**
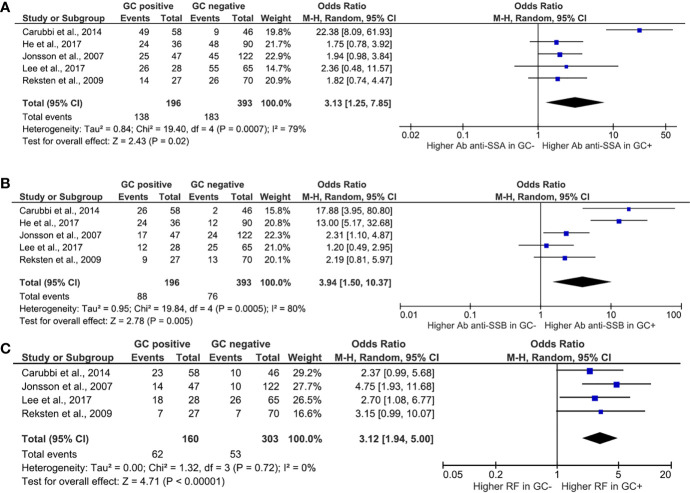
Meta-analysis of the presence of anti-SSA **(A)**, anti-SSB **(B)**, and rheumatoid factor **(C)** between patients with Sjögren’s syndrome with or without ectopic germinal center. The size of squares is proportional to the weight of each study. Horizontal lines indicate the 95% CI of each study; diamond, the pooled estimate with 95% CI; N, the number of persons at baseline; and OR, odds ratio; GC, germinal center; RF, rheumatoid factor.

Among clinical features, the presence of ectopic GC and its association with glandular swelling (**Figure 2A**), arthritis (**Figure 2B**), and RP (**Figure 2C**) were explored. Ectopic GCs presence was not associated with a significant increase in the odds ratio for all the explored variables. As far as the glandular swelling is concerned, the lack of association was maintained after the “leave one out” test with a significant reduction in the heterogeneity, from 50 to 0%, confirming the reliability of the results. Concerning the serological features, ectopic GCs presence accounted for an increased ratio of anti-SSA, anti-SSB autoantibodies, and RF presence. The ectopic GCs presence at histologic evaluation increased the anti-SSA presence probability (OR = 3.13, 95% CI: 1.25–7.85, p = 0.02), as shown in **Figure 3A**. A significant heterogeneity was observed among the studies (p for heterogeneity = 0.0007, I^2^ = 79%), which was mainly observed in the study of Carubbi et al. (20). However, removing this study from the analysis did not change the outcome confirming the association (**Figure 3B**). Ectopic GCs presence at the histologic evaluation increased the probability of anti-SSB antibodies’ presence (OR = 3.94, 95% CI: 1.50–10.37, p = 0.0005, I^2^ = 80%), and also this outcome was maintained during the “leave one out” test. Finally, ectopic GCs presence increased the probability of RFs presence in pSS patients’ sera (OR = 3.12, 95% CI: 1.94–5.00, p < 0.00001, I^2^ = 0%), without any heterogeneity among the selected studies (**Figure 3C**).

### Focus Score Influence on Clinical and Serological Features in pSS

Due to the different classification systems based on FS used in the retrieved papers, quantitative analysis was not possible. Thus we summarized the available data. Wise et al. in 1993 explored the correlation between pSS clinical features and mSGB results: this study, for the first time, pointed out the lack of correlation between FS and clinical features but unmasked the link between FS and serological markers. In fact, in this cohort, among the selected clinical features (dry eyes, Shirmer test, dry mouth, salivary swelling, pulmonary findings, renal findings, GI findings, thyroid disease, cutaneous lesions, adenopathy, neurologic findings), only the salivary swelling was significantly present in patients with FS >1. On the other hand, serologic findings, defined as ANA, anti-SSA, anti-SSB, and RFs presence, were significantly increased in patients with FS >1, when they were considered individually or as a group (23). Reksten et al., in 2009, did not confirm these results, showing that patients in the FS− group had a higher frequency of both anti-SSA and anti-SSB antibodies when compared to patients in the FS+ group, which may be related to the specific inclusion criteria used in the study (19). Successively, Daniels et al. explored the associations between SG histopathologic changes and phenotypic features in 1,726 pSS patients from the database of the Sjögren’s International Collaborative Clinical Alliance (SICCA). They found that FS >1 was significantly associated with serum anti-SSA and anti-SSB positivity, RF, but not with symptoms of dry mouth and/or dry eyes. Patients with positive anti-SSA/SSB were nine times (95% CI: 7.4–11.9) more likely to have a focus score of >1 than those without anti-SSA/SSB. Of note, patients with an unstimulated whole salivary flow rate of <0.1 ml/min were two times (95% CI 1.7–2.8) more likely to have a focus score of >1 than those with a higher flow rate (25). In 2015, Carubbi et al. confirmed the variability of pSS disease spectrum among patients with different FSs. In their experience, higher FS values were associated with a significant higher frequency of salivary gland swelling and lymphoma. Furthermore, reduction of C4, hypergammaglobulinemia, circulating monoclonal component, and double association anti-SSA and anti-SSB were more common in patients with FS ≥1 (24). The main characteristics of patients enrolled in these studies are summarized in **Tables 3**, **4**.

### Salivary Gland Biopsy Prognostic Role

In the 2014, Risselada et al. explored the prognostic role of mSGB in pSS patients’ follow-up. In a retrospective study, they analyzed the prognostic value of FS and the percentages of IgA+, IgM+, and IgG+ plasma cells in mSGBs on disease outcomes. Their results showed that mean FS was significantly higher in patients developing non-Hodgkin lymphoma (NHL) (3.0 ± 0.894 *vs* 2.25 ± 1.086; p = 0.021), and FS ≥3 foci had a positive predictive value of 16% for NHL and a negative predictive value of 98%. Only FS ≥3 contributed significantly and independently to NHL development in a standard multiple regression model (26). The prognostic value of FS on lymphoma development was confirmed in 2015 by Carubbi et al. In fact, in a multivariate analysis, they showed that patients with higher FS had a higher risk of developing lymphoma (OR = 1.314, 95% CI: 1.090–1.585, p = 0.004) (24). In another retrospective study, a FS ≥4 was, together with age and male gender, a risk factor for interstitial lung disease (ILD) development in pSS patients (OR = 3.954, 95% CI: 1.423–10.987, p = 0,008) (27).

## Discussion

mSGB is a cornerstone in pSS diagnosis, but to date, its role is underestimated in the follow-up of pSS patients, and behind its valuable role in pSS classification criteria, its potential use in different clinimetric settings is still poorly recognized. Our SLR pointed out the limited number of studies that explored the association between histologic scores and pSS clinical presentation and prognosis, confirming what was already reported (28, 29). Furthermore, in the analyzed studies, different standards and different definitions were used to evaluate the results of mSGBs and any study that independently selected the clinical features of disease presentation, making challenging the comparison of the results and the quantitative analysis. Furthermore, mSGB interpretation and GCs detection could be influenced by the pathologist’s experience.

Here, analyzing data derived from 589 pSS patients enrolled in five studies, we did not find any association between ectopic GCs presence at diagnosis and salivary gland swelling, arthritis, and RP presence. On the other hand, patients with ectopic GCs presence, in their mSGBs, showed a higher OR for anti-SSA, anti-SSB, and RF positivity. B lymphocyte accumulation in pSS salivary glands is a key feature of the disease, and ectopic GC structures promote their chronic stimulation and activation. B lymphocytes producing autoantibodies were described at the borders of ectopic GCs (30). To date, the sites involved in autoantibodies’ production during pSS are not fully elucidated, but affected salivary glands seem to contribute to the production. The presence of plasma cells with intracytoplasmic immunoglobulins with anti-SSA activity in the mSGs, the finding of autoreactive B lymphocytes in the ectopic GC structures, and the evidence of autoantibodies in the saliva contribute to support this hypothesis (30, 31). Furthermore, *in vitro* studies demonstrated the ability of epithelial salivary gland cells in the exposure of Ro60/TRIM, Ro52/TROVE2, and La/SSB during their death, fueling the autoimmune response (32, 33). Our data mirrors what has been already reported in the literature, in which ectopic GCs presence in mSGBs, hosting B lymphocyte chronic activation, selection, and affinity maturation (34), may be associated with serological presentation and autoimmune phenomena activation, characterizing a pSS phenotype with potentially a more severe disease. Furthermore, FLS and ectopic GC being a continuum in the inflammatory infiltrate characterizing the mSGs of pSS patients, these results reinforce the reported association between FS and serologic features (23–25), lymphoma (24, 26), and ILD development (27).

To our knowledge, this systematic review and meta-analysis is the first report to provide a comprehensive analysis of the clinical findings and laboratory abnormalities associated with histologic markers in pSS patients. Despite the low number of high-quality published data, after combining all the studies using the conservative random-effects model, the obtained results may be considered robust and reliable.

We are aware that our results may be influenced by the lack of randomized control studies as well as by the small number of available studies, mainly retrospective in nature. Therefore, the overall generalizability of our meta-analysis results may be outdated in the future when randomized studies will be published.

In conclusion, pSS patients with ectopic GC, despite exhibiting similar glandular dysfunction and clinical presentation with the patients without ectopic GCs presence, show different features across laboratory parameters, which are mainly related to B lymphocyte hyperactivity. The formation of ectopic GC within the salivary glands of pSS patients may be an important step in the process leading to lymphocytic sialadenitis. Although our study failed to identify any significant associations between the presence of ectopic GC and clinical features, ectopic GC positive pSS patients do exhibit distinct serological features, highlighting the importance of mSGBs behind pSS diagnosis. Studies specifically designed are necessary to confirm these results in larger cohorts to definitively assess the importance of these laboratory abnormalities and their relevance for the clinical outcomes.

## Data Availability Statement

The original contributions presented in the study are included in the article/**Supplementary Material**. Further inquiries can be directed to the corresponding author.

## Author Contributions

OB, PR, PC, and RG contributed to conception and design of the study. SD’A performed the statistical analysis. OB and PR wrote the first draft of the manuscript. PB, LN, and AM wrote sections of the manuscript and participated in the literature review. All authors contributed to the article and approved the submitted version.

## Conflict of Interest

The authors declare that the research was conducted in the absence of any commercial or financial relationships that could be construed as a potential conflict of interest.
