# Supplementary material for: Association Between Minor Salivary Gland Biopsy During Sjӧgren’s Syndrome and Serologic Biomarkers: A Systematic Review and Meta-Analysis
Source: Front Immunol. 2021 Jun 11;12:686457. doi: 10.3389/fimmu.2021.686457 (PMC8226119; doi:10.3389/fimmu.2021.686457)
Supplement: Supplementary file 1 [file Table_1.docx]

**Supplementary Table 1. Papers excluded from the analysis with the main reason.**

| **First Author** | **Year** | **Title** | **Main reason for exclusion** |
| --- | --- | --- | --- |
| Sepúlveda, D. | 2018 | Impaired IRE1α/XBP-1 pathway associated to DNA methylation might contribute to salivary gland dysfunction in Sjögren's syndrome patients. | out of interest |
| Zandbelt, M. | 2004 | Etanercept in the treatment of patients with primary Sjögren's syndrome: a pilot study. | out of interest |
| Zheng, Li. | 2010 | Expression of Toll-like receptors 7, 8, and 9 in primary Sjögren's syndrome. | out of interest |
| Katsifis, G. | 2009 | Systemic and local interleukin-17 and linked cytokines associated with Sjögren's syndrome immunopathogenesis. | out of interest |
| Steinfeld, S. | 2002 | Treatment with infliximab restores normal aquaporin 5 distribution in minor salivary glands of patients with Sjögren's syndrome. | out of interest |
| Szyszko, E. | 2011 | Salivary glands of primary Sjögren's syndrome patients express factors vital for plasma cell survival. | out of interest |
| Lucchesi, D. | 2020 | The use of digital image analysis in the histological assessment of Sjögren's syndrome salivary glands improves inter-rater agreement and facilitates multicentre data harmonisation. | out of interest |
| Thrane, P. | 1988 | Differential expression of epithelial MHC class II determinants (HLA-DR, -DP, and -DQ) and increased class I expression in inflamed salivary glands. | out of interest |
| Miyazaki, K. | 2005 | Analysis of in vivo role of alpha-fodrin autoantigen in primary Sjogren's syndrome. | out of interest |
| Skarstein, K. | 2016 | Adipose tissue is prominent in salivary glands of Sjögren's syndrome patients and appears to influence the microenvironment in these organs. | out of interest |
| de Azevedo, B. | 2005 | Evaluation of sialometry and minor salivary gland biopsy in classification of Sjögren's Syndrome patients. | not in English |
| Skopouli, F. | 1998 | Association of mast cells with fibrosis and fatty infiltration in the minor salivary glands of patients with Sjögren's syndrome. | out of interest |
| Li, X. | 2007 | Expression of forkhead box protein P3 in peripheral blood and salivary gland of patients with Sjogren's syndrome. | not in English |
| Skopouli, F. | 1991 | T cell subpopulations in the labial minor salivary gland histopathologic lesion of Sjögren's syndrome. | out of interest |
| Alunno, A. | 2015 | Telocytes in minor salivary glands of primary Sjögren's syndrome: association with the extent of inflammation and ectopic lymphoid neogenesis. | out of interest |
| Mason, G. | 2003 | Salivary gland expression of transforming growth factor beta isoforms in Sjogren's syndrome and benign lymphoepithelial lesions. | out of interest |
| Llamas-Gutierrez, F. | 2014 | Histopathological environment besides the focus score in Sjögren's syndrome. | out of interest |
| Steinfeld, S. | 2001 | Abnormal distribution of aquaporin-5 water channel protein in salivary glands from Sjögren's syndrome patients. | out of interest |
| Ek, M. | 2006 | Increased extracellular levels of the novel proinflammatory cytokine high mobility group box chromosomal protein 1 in minor salivary glands of patients with Sjögren's syndrome. | out of interest |
| Kucuk, U. | 2018 | Histopathological differences between primary Sjögren's syndrome and Sjögren's syndrome accompanied by scleroderma. | out of interest |
| Tarpley, T. | 1974 | Minor salivary gland involvement in Sjögren's syndrome. | out of interest |
| Fox, R. | 1983 | Characterization of the phenotype and function of lymphocytes infiltrating the salivary gland in patients with primary Sjogren syndrome. | out of interest |
| Steinfeld, S. | 2000 | Prolactin up-regulates cathepsin B and D expression in minor salivary glands of patients with Sjögren's syndrome. | out of interest |
| Lahiri, A. | 2014 | Specific forms of BAFF favor BAFF receptor-mediated epithelial cell survival. | out of interest |
| Leroy, J. | 1992 | Follow up study of labial salivary gland lesions in primary Sjögren's syndrome. | out of interest |
| Steinfeld, S. | 2000 | Big prolactin 60 kDa is overexpressed in salivary glandular epithelial cells from patients with Sjögren's syndrome. | out of interest |
| Cristiano, A. | 2017 | CD4 T lymphocyte autophagy is upregulated in the salivary glands of primary Sjögren's syndrome patients and correlates with focus score and disease activity. | out of interest |
| Zandbelt, M. | 2001 | Reversibility of histological and immunohistological abnormalities in sublabial salivary gland biopsy specimens following treatment with corticosteroids in Sjögren's syndrome. | case report |
| Konttinen, Y. | 1990 | Mast cells in the labial salivary glands of patients with Sjögren's syndrome: a histochemical, immunohistochemical, and electron microscopical study. | out of interest |
| Guellec, D. | 2013 | Diagnostic value of labial minor salivary gland biopsy for Sjögren's syndrome: a systematic review. | review |
| Apostolou, E. | 2016 | Expression of type III interferons (IFNλs) and their receptor in Sjögren's syndrome. | out of interest |
| Konttinen, Y. | 1990 | Localization of lysozyme mRNA in the labial salivary glands by in situ hybridization in Sjögren's syndrome. | out of interest |
| Poduval, P. | 2009 | Abnormal basement membrane type IV collagen alpha-chain composition in labial salivary glands in Sjögren's syndrome. | out of interest |
| Benucci, M. | 2003 | Association between serum amyloid A (SAA) in salivary glands and high levels of circulating beta 2-microglobulin in patients with Sjögren syndrome. | not in English |
| Manganelli, P. | 1997 | Quantitative analysis of apoptosis and bcl-2 in Sjögren's syndrome. | out of interest |
| Bolstad, A. | 2003 | Increased salivary gland tissue expression of Fas, Fas ligand, cytotoxic T lymphocyte-associated antigen 4, and programmed cell death 1 in primary Sjögren's syndrome. | out of interest |
| McNamara, N. | 2014 | Establishing PAX6 as a biomarker to detect early loss of ocular phenotype in human patients with Sjögren's syndrome. | out of interest |
| Zuo, J. | 2016 | Muscarinic type 3 receptor autoantibodies are associated with anti-SSA/Ro autoantibodies in Sjögren's syndrome. | out of interest |
| Larsson, A. | 2001 | Ku protein and DNA strand breaks in lip glands of normal and primary Sjögren's syndrome subjects: lack of correlation with apoptosis. | out of interest |
| Ittah, M. | 2006 | B cell-activating factor of the tumor necrosis factor family (BAFF) is expressed under stimulation by interferon in salivary gland epithelial cells in primary Sjögren's syndrome. | out of interest |
| Caporali, R. | 2008 | Safety and usefulness of minor salivary gland biopsy: retrospective analysis of 502 procedures performed at a single center. | out of interest |
| Sisto, M. | 2010 | Expression of pro-inflammatory TACE-TNF-α-amphiregulin axis in Sjögren's syndrome salivary glands. | out of interest |
| Trivedi, A. | 2021 | Employing immunohistochemical staining to labial minor salivary gland biopsies from patients with Sjogren's syndrome increases diagnostic certainty. | out of interest |
| Törnwall, J. | 1997 | Protein kinase C expression in salivary gland acinar epithelial cells in Sjögren's syndrome. | letter |
| Lucchesi, D. | 2020 | Impaired Interleukin-27-Mediated Control of CD4+ T Cell Function Impact on Ectopic Lymphoid Structure Formation in Patients With Sjögren's Syndrome. | out of interest |
| Baer, A. | 2016 | Association of Anticentromere Antibodies With More Severe Exocrine Glandular Dysfunction in Sjögren's Syndrome: Analysis of the Sjögren's International Collaborative Clinical Alliance Cohort. | out of interest |
| Pringle, S. | 2019 | Salivary Gland Stem Cells Age Prematurely in Primary Sjögren's Syndrome. | out of interest |
| Tavoni, A. | 2012 | Minor salivary gland biopsy and Sjögren's syndrome: comparative analysis of biopsies among different Italian rheumatologic centers. | out of interest |
| Blokland, S. | 2020 | Epigenetically quantified immune cells in salivary glands of Sjögren's syndrome patients: a novel tool that detects robust correlations of T follicular helper cells with immunopathology. | out of interest |
| Sarioğlu, S. | 2016 | Minor salivary gland evaluation: Sjögren's syndrome. | out of interest |
| Markopoulos, A. | 2000 | Immunohistochemical detection of insulin-like growth factor-I in the labial salivary glands of patients with Sjögren's syndrome. | out of interest |
| Stone, D. | 2017 | Effect of Tobacco Smoking on The Clinical, Histopathological, and Serological Manifestations of Sjögren's Syndrome. | out of interest |
| Mieliauskaite, D. | 2012 | Expression of IL-17, IL-23 and their receptors in minor salivary glands of patients with primary Sjögren's syndrome. | out of interest |
| Qiao, L. | 2015 | The Clinical Characteristics of Primary Sjogren's Syndrome With Neuromyelitis Optica Spectrum Disorder in China: A STROBE-Compliant Article. | out of interest |
| Nanke, Y. | 2016 | Detection of IFN-γ+IL-17+ cells in salivary glands of patients with Sjögren's syndrome and Mikulicz's disease: Potential role of Th17•Th1 in the pathogenesis of autoimmune diseases. | out of interest |
| Sisto, M. | 2017 | Abnormal distribution of AQP4 in minor salivary glands of primary Sjögren's syndrome patients. | out of interest |
| Stott, D. | 1998 | Antigen-driven clonal proliferation of B cells within the target tissue of an autoimmune disease. The salivary glands of patients with Sjögren's syndrome. | out of interest |
| Kong, L. | 1997 | Fas and Fas ligand expression in the salivary glands of patients with primary Sjögren's syndrome. | out of interest |
| Adler, S. | 2013 | Evaluation of histologic, serologic, and clinical changes in response to abatacept treatment of primary Sjögren’s syndrome: a pilot study. | out of interest |
| Schuurman, H. | 1989 | Epstein-Barr virus in the sublabial salivary gland in Sjögren's syndrome. | out of interest |
| Haacke, E. | 2017 | Abatacept treatment of patients with primary Sjögren's syndrome results in a decrease of germinal centres in salivary gland tissue. | out of interest |
| Katz, J. | 2003 | IGF-1 and insulin receptor expression in the minor salivary gland tissues of Sjögren's syndrome and mucoceles--immunohistochemical study. | out of interest |
| Takahashi, H. | 1996 | Immunohistochemical findings of arterial fibrinoid necrosis in major and lingual minor salivary glands of primary Sjögren's syndrome. | out of interest |
| Lee, J. | 2018 | JAK-1 Inhibition Suppresses Interferon-Induced BAFF Production in Human Salivary Gland: Potential Therapeutic Strategy for Primary Sjögren's Syndrome. | out of interest |
| Nicaise, C. | 2017 | Phagocyte-specific S100A8/A9 is upregulated in primary Sjögren's syndrome and triggers the secretion of pro-inflammatory cytokines in vitro. | out of interest |
| Maślińska, M. | 2019 | Usefulness of rheumatoid factor as an immunological and prognostic marker in PSS patients. | out of interest |
| Bautista-Vargas, M. | 2020 | Minor salivary gland biopsy: Its role in the classification and prognosis of Sjögren's syndrome. | review |
| Batbayar, B. | 2002 | Changes of the nerve fibers innervating the minor salivary glands in Sjögren syndrome. | not in English |
| Fox, R. | 1992 | Laboratory evaluation of patients with Sjögren's syndrome. | out of interest |
| Billings, M. | 2016 | Significance and Implications of Patient-reported Xerostomia in Sjögren's Syndrome: Findings From the National Institutes of Health Cohort. | out of interest |
| Xu, K. | 1997 | Biopsy of labial salivary glands and lacrimal glands in the diagnosis of Sjögren's syndrome. | not in English |
| Katsiougiannis, S. | 2006 | Salivary gland epithelial cells: a new source of the immunoregulatory hormone adiponectin. | out of interest |
| Baldini, C. | 2015 | Salivary gland ultrasonography: a highly specific tool for the early diagnosis of primary Sjögren's syndrome. | out of interest |
| Andonopoulos, A. | 1989 | Sjögren's syndrome in rheumatoid arthritis and progressive systemic sclerosis. A comparative study. | out of interest |
| Kang, E. | 2011 | Salivary cytokine profiles in primary Sjögren's syndrome differ from those in non-Sjögren sicca in terms of TNF-α levels and Th-1/Th-2 ratios. | out of interest |
| Kim, J. | 2016 | A validated method of labial minor salivary gland biopsy for the diagnosis of Sjögren's syndrome. | out of interest |
| Sisto, M. | 2018 | Reduced myofilament component in primary Sjögren's syndrome salivary gland myoepithelial cells. | out of interest |
| Barrera, M. | 2016 | Pro-inflammatory cytokines enhance ERAD and ATF6α pathway activity in salivary glands of Sjögren's syndrome patients. | out of interest |
| Vettori, S. | 2016 | Serum CXCL4 increase in primary Sjögren's syndrome characterizes patients with microvascular involvement and reduced salivary gland infiltration and lymph node involvement. | out of interest |
| Nakamura, H. | 2018 | Detection of human T lymphotropic virus type-I bZIP factor and tax in the salivary glands of Sjögren's syndrome patients. | out of interest |
| Bergum, B. | 2016 | Antibodies against carbamylated proteins are present in primary Sjögren's syndrome and are associated with disease severity. | out of interest |
| Oxholm, P. | 1987 | Platelet involvement in salivary gland inflammation in patients with primary Sjögren's syndrome. | out of interest |
| Wang, X. | 2007 | Immunopathology of labial glands of patients with primary Sjögren's syndrome. | out of interest |
| Beroukas, D. | 2002 | Up-regulation of M3-muscarinic receptors in labial salivary gland acini in primary Sjögren's syndrome. | out of interest |
| Boumba, D. | 1995 | Cytokine mRNA expression in the labial salivary gland tissues from patients with primary Sjögren's syndrome. | out of interest |
| Li, X. | 2007 | Expression of CD4+ CD25+ regulatory T cells in peripheral blood and salivary gland of patients with primary Sjögren's syndrome. | not in English |
| de Wilde, P. | 1996 | Aberrant expression pattern of the SS-B/La antigen in the labial salivary glands of patients with Sjögren's syndrome. | out of interest |
| Mariette, X. | 2004 | Inefficacy of infliximab in primary Sjögren's syndrome: results of the randomized, controlled Trial of Remicade in Primary Sjögren's Syndrome (TRIPSS). | out of interest |
| Hedfords, E. | 1990 | Variation of MHC class I and II antigen expression in relation to lymphocytic infiltrates and interferon-gamma positive cells. | out of interest |
| Liu, X. | 2000 | G-protein signaling abnormalities mediated by CD95 in salivary epithelial cells. | out of interest |
| Shi, H. | 2016 | Long non-coding RNA expression profile in minor salivary gland of primary Sjögren's syndrome. | out of interest |
| Egerer, T. | 2006 | Tissue-specific up-regulation of the proteasome subunit beta5i (LMP7) in Sjögren's syndrome. | out of interest |
| Segerberg-Konttinen, M. | 1986 | Focus score in the diagnosis of Sjögren's syndrome. | out of interest |
| Morbini, P. | 2005 | Multilevel examination of minor salivary gland biopsy for Sjogren's syndrome significantly improves diagnostic performance of AECG classification criteria. | out of interest |
| Scardina, G. | 2007 | Diagnostic evaluation of serial sections of labial salivary gland biopsies in Sjögren's syndrome. | out of interest |
| Sisto, M. | 2015 | The metalloproteinase ADAM17 and the epidermal growth factor receptor (EGFR) signaling drive the inflammatory epithelial response in Sjögren's syndrome. | out of interest |
| Ohlsson, M. | 2001 | Fas-induced apoptosis is a rare event in Sjögren's syndrome. | out of interest |
| Kaneda, Y. | 2009 | Localization of antimicrobial peptides human beta-defensins in minor salivary glands with Sjögren's syndrome. | out of interest |
| Konttinen, Y. | 1998 | Matrix metalloproteinase (MMP)-9 type IV collagenase/gelatinase implicated in the pathogenesis of Sjögren's syndrome. | out of interest |
| van Woerkom, J. | 2007 | Safety and efficacy of leflunomide in primary Sjögren's syndrome: a phase II pilot study. | out of interest |
| Aqrawi, L. | 2014 | Ductal epithelial expression of Ro52 correlates with inflammation in salivary glands of patients with primary Sjögren's syndrome. | out of interest |
| Kop'eva, T. | 1994 | Morphology of the minor salivary glands in Sjogren's disease. | not in English |
| Deng, F. | 2016 | Association of BAFF and IL-17A with subphenotypes of primary Sjögren's syndrome. | out of interest |
| Fei, Y. | 2013 | Importance of salivary gland focus score in the diagnosis of Sjögren's syndrome. | not in English |
| Sisto, M. | 2014 | Neovascularization is prominent in the chronic inflammatory lesions of Sjögren's syndrome. | out of interest |
| Thrane, P. | 1993 | Increased epithelial expression of HLA-DQ and HLA-DP molecules in salivary glands from patients with Sjögren's syndrome compared with obstructive sialadenitis. | out of interest |
| Bosello, S. | 2016 | Thymosin β(4) and β(10) in Sjögren's syndrome: saliva proteomics and minor salivary glands expression. | out of interest |
| Szabo, K. | 2016 | Follicular helper T cells may play an important role in the severity of primary Sjögren's syndrome. | out of interest |
| Saito, I. | 1993 | Expression of cell adhesion molecules in the salivary and lacrimal glands of Sjogren's syndrome. | out of interest |
| Kalogirou, E. | 2018 | Ductal cells of minor salivary glands in Sjögren's syndrome express LINE-1 ORF2p and APOBEC3B. | out of interest |
| Baldini, C. | 2013 | The P2X7 receptor-inflammasome complex has a role in modulating the inflammatory response in primary Sjögren's syndrome. | out of interest |
| Molina, C. | 2006 | Basal lamina disorganisation of the acini and ducts of labial salivary glands from patients with Sjogren's syndrome: association with mononuclear cell infiltration. | out of interest |
| Xu, K. | 1996 | Biopsy of labial salivary glands and lacrimal glands in the diagnosis of Sjögren's syndrome. | out of interest |
| Bodeutsch, C. | 1992 | Quantitative immunohistologic criteria are superior to the lymphocytic focus score criterion for the diagnosis of Sjögren's syndrome. | out of interest |
| Wen, S. | 1996 | Association of Epstein-Barr virus (EBV) with Sjögren's syndrome: differential EBV expression between epithelial cells and lymphocytes in salivary glands. | out of interest |
| Huguet, P. | 1995 | Sjögren's syndrome: histologic and immunohistochemical study. | out of interest |
| Kovács, L. | 2008 | Demonstration of autoantibody binding to muscarinic acetylcholine receptors in the salivary gland in primary Sjögren's syndrome. | out of interest |
| Katz, J. | 2004 | Receptor of advanced glycation end product (RAGE) expression in the minor salivary glands of patients with Sjögren's syndrome: a preliminary study. | out of interest |
| Soejima, K. | 2007 | Activation of MKK4 (SEK1), JNK, and c-Jun in labial salivary infiltrating T cells in patients with Sjögren's syndrome. | out of interest |
| Nakamura, H. | 2007 | EGF activates PI3K-Akt and NF-kappaB via distinct pathways in salivary epithelial cells in Sjögren's syndrome. | out of interest |
| Mavragani, C. | 2016 | Expression of Long Interspersed Nuclear Element 1 Retroelements and Induction of Type I Interferon in Patients With Systemic Autoimmune Disease. | out of interest |
| Furuzawa-Carballeda, J. | 2014 | Differential cytokine expression and regulatory cells in patients with primary and secondary Sjögren's syndrome. | out of interest |
| Sisto, M. | 2016 | Interleukin-15 as a potential new target in Sjögren's syndrome-associated inflammation. | out of interest |
| Hua, H. | 1995 | An immunohistochemical study of HLA-DR expression in salivary glands from patients with Sjogren's syndrome | not in English |
| Weng, X. | 2018 | The role of RORα in salivary gland lesions in patients with primary Sjögren's syndrome. | out of interest |
| Kim, J. | 2018 | Salivary gland ultrasonography findings are associated with clinical, histological, and serologic features of Sjögren's syndrome. | out of interest |
| de Wilde, P. | 1986 | Morphometry in the diagnosis of Sjögren's syndrome. | out of interest |
| Kang, Y. | 2013 | Abnormal expression of CytC in the labial gland of patients with primary Sjoigren's syndrome. | not in English |
| Yokogawa, N. | 2014 | Comparison of labial minor salivary gland biopsies from childhood Sjögren syndrome and age-matched controls. | out of interest |
| Nakamura, H. | 1999 | Expression of mitogen activated protein kinases in labial salivary glands of patients with Sjögren's syndrome. | out of interest |
| Konttinen, Y. | 2000 | Mast cell derangement in salivary glands in patients with Sjögren's syndrome. | out of interest |
| Konttinen, Y. | 1981 | In situ characterization of the cellular infiltrate in labial and palatine glands in Sjögren's syndrome. | out of interest |
| Fragoulis, G. | 2016 | Analysis of the cell populations composing the mononuclear cell infiltrates in the labial minor salivary glands from patients with rheumatoid arthritis and sicca syndrome. | out of interest |
| Le Charpentier, Y. | 1994 | Histopathologic lesions of the accessory salivary glands in Gougerot-Sjögren syndrome: re-evaluation of the diagnostic criteria of Chisholm and Mason and of Chometter et al. | not in English |
| Friberg, B. | 1988 | Salivary kallikrein in Sjögren's syndrome. | out of interest |
| Smith, C. | 1992 | Labial salivary gland histopathology and autoantibodies in Sjögren's syndrome and other connective tissue diseases. | letter |
| Andretta, M. | 1995 | Diagnostic value of labial salivary biopsy in Sjogren's syndrome: report of 182 cases. | not in English |
| Konttinen, Y. | 1995 | Neutral endopeptidase (EC 3.4.24.11) in labial salivary glands in healthy controls and in patients with Sjögren's syndrome. | out of interest |
| Koski, H. | 1997 | Epidermal growth factor, transforming growth factor-alpha, and epidermal growth factor receptor in labial salivary glands in Sjögren's syndrome. | out of interest |
| Le Charpentier, Y. | 1994 | What can be expected of the microscopic study of a biopsy of the accessory salivary glands? An argument for a new approach: "D.D." (diagnosis, destruction). | not in English |
| Spadaro, A. | 2001 | Soluble interleukin-2 receptor in Sjögren's syndrome: relation to main serum immunological and immunohistochemical parameters. | out of interest |
| Aksoy, T. | 2012 | Correlations between histopathologic and scintigraphic parameters of salivary glands in patients with Sjögren's syndrome. | out of interest |
| Prochorec-Sobieszek, M. | 2004 | Histopathological and immunohistochemical analysis of lymphoid follicles in labial salivary glands in primary and secondary Sjögren's syndrome. | out of interest |
| Brito-Zerón, P. | 2018 | Phenotyping Sjögren's syndrome: towards a personalised management of the disease. | review |
| Cleary, K. | 1990 | Biopsy of the lip and Sjögren's syndrome. | review |
| de Wilde, P | 1984 | Multinucleate giant cells in sublabial salivary gland tissue in Sjögren's syndrome. A diagnostic pitfall. | out of interest |
| Cleland-Zamudio, S. | 1993 | Pathology of labial salivary gland cellular aggregates in Sjögren's syndrome. | out of interest |
| Pèrez, P, | 2005 | Increased acinar damage of salivary glands of patients with Sjögren's syndrome is paralleled by simultaneous imbalance of matrix metalloproteinase 3/tissue inhibitor of metalloproteinases 1 and matrix metalloproteinase 9/tissue inhibitor. | out of interest |
| Kilpi, A. | 1988 | Characterization of mononuclear cells of inflammatory infiltrates in oral tissues. A histochemical and immunohistochemical study of labial salivary glands in Sjögren's syndrome and of oral lesions in systemic lupus erythematosus and in lichen planus. | out of interest |
| Sidagis, J. | 1997 | Expression of glycoconjugates in normal and Sjögren's syndrome labial glands. | out of interest |
| Koski, H. | 1995 | Transforming growth factor beta 2 in labial salivary glands in Sjögren's syndrome. | out of interest |
| Ciccia, F. | 2015 | Interleukin-36α axis is modulated in patients with primary Sjögren's syndrome. | out of interest |
| Caretto, A. | 1995 | An immunohistochemical study of immunological phenomena in minor salivary glands in patients with Sjögren's syndrome. | out of interest |
| Leehan, K. | 2017 | Fatty infiltration of the minor salivary glands is a selective feature of aging but not Sjögren's syndrome. | out of interest |
| Törnwall, J. | 1994 | Distribution of vasoactive intestinal peptide (VIP) and its binding sites in labial salivary glands in Sjögren's syndrome and in normal controls. | out of interest |
| Larsson, A. | 2015 | Immunohistochemistry of the B-cell component in lower lip salivary glands of Sjögren's syndrome and healthy subjects. | out of interest |
| Bombardieri, M. | 2004 | Increased circulating levels and salivary gland expression of interleukin-18 in patients with Sjögren's syndrome: relationship with autoantibody production and lymphoid organization of the periductal inflammatory infiltrate. | out of interest |
| Xanthou, G. | 1999 | CD4 cytotoxic and dendritic cells in the immunopathologic lesion of Sjögren's syndrome. | out of interest |
| Konttinen, Y. | 1994 | Collagenase in Sjögren's syndrome. | out of interest |
| Fava-De-Moraes, F. | 1978 | Histochemical study of labial salivary glands in Sjögren's syndrome. | out of interest |
| Ozaki, Y. | 2010 | Decrease of blood dendritic cells and increase of tissue-infiltrating dendritic cells are involved in the induction of Sjögren's syndrome but not in the maintenance. | out of interest |
| Matsumura, R. | 1996 | Decrease of blood dendritic cells and increase of tissue-infiltrating dendritic cells are involved in the induction of Sjögren's syndrome but not in the maintenance. | out of interest |
| Zeher, M. | 1994 | Fibrinolysis-resistant fibrin deposits in minor labial salivary glands of patients with Sjögren's syndrome. | out of interest |
| Dinescu, S. | 2017 | Histopathological and immunohistochemical profile in primary Sjögren's syndrome. | out of interest |
| Nakamura, H. | 2000 | Expression and function of X chromosome-linked inhibitor of apoptosis protein in Sjögren's syndrome. | out of interest |
| Bodeutsch, C. | 1991 | Diagnostic and prognostic value of quantitative immunohistological examination of lip biopsy in Sjögren's syndrome. | case report |
| Hall, J. | 2015 | Molecular Subsetting of Interferon Pathways in Sjögren's Syndrome. | out of interest |
| Ohlsson, M. | 2002 | CD40, CD154, Bax and Bcl-2 expression in Sjögren's syndrome salivary glands: a putative anti-apoptotic role during its effector phases. | out of interest |
| Lombardi, T. | 2011 | Absence of up-regulation for a proliferation-inducing ligand in Sjögren's sialadenitis lesions. | out of interest |
| Skopouli, F. | 1992 | c-myc mRNA expression in minor salivary glands of patients with Sjögren's syndrome. | out of interest |
| Kapsogeorgou, E. | 2019 | Predictive markers of lymphomagenesis in Sjögren's syndrome: From clinical data to molecular stratification. | review |
| Skarstein, K. | 2019 | Autoantigen-specific B cells and plasma cells are prominent in areas of fatty infiltration in salivary glands of patients with primary Sjögren's syndrome. | out of interest |
| Costa, S. | 2015 | Reliability of histopathological salivary gland biopsy assessment in Sjögren's syndrome: a multicentre cohort study. | out of interest |
| Amft, N. | 2001 | Ectopic expression of the B cell-attracting chemokine BCA-1 (CXCL13) on endothelial cells and within lymphoid follicles contributes to the establishment of germinal center-like structures in Sjögren's syndrome. | out of interest |
| Guggino, G. | 2018 | Interleukin-25 Axis Is Involved in the Pathogenesis of Human Primary and Experimental Murine Sjögren's Syndrome. | out of interest |
| Nakamura, H. | 1999 | Expression of CD40/CD40 ligand and Bcl-2 family proteins in labial salivary glands of patients with Sjogren's syndrome. | out of interest |
| Risselada, A. | 2015 | Lymphocytic focus score as a prognostic tool. | letter |
| Haacke, E. | 2017 | FcRL4+ B-cells in salivary glands of primary Sjögren's syndrome patients. | out of interest |
| Barone, F. | 2008 | CXCL13, CCL21, and CXCL12 expression in salivary glands of patients with Sjögren's syndrome and MALT lymphoma: Association with reactive and malignant areas of lymphoid organization. | out of interest |
| Alpert, S. | 1994 | Expression of granzyme A in salivary gland biopsies from patients with primary Sjogren’s syndrome | out of interest |
| Bikker, A. | 2015 | Increased interleukin (IL)-7Rα expression in salivary glands of patients with primary Sjögren’s syndrome is restricted to T cells and correlates with IL-7 expression, lymphocyte numbers and activity. | out of interest |
| Castro,I. | 2012 | Decreased salivary sulphotransferase activity correlated with inflammation and autoimmunity parameters in Sjogren’s syndrome patients | out of interest |
| Erasan, F. | 2016 | Leptin and leptin receptors in salivary glands of primary Sjögren’s syndrome. | out of interest |
| Giovelli, R. | 2015 | Clinical characteristics and biopsy accuracy in suspected cases of Sjögren’s syndrome referred to labial salivary gland biopsy. | out of interest |
| Jonhsen, S. | 2015 | Low Protein A20 in Minor Salivary Glands is Associated with Lymphoma in Primary Sjogren’s Syndrome. | out of interest |
| Jonsson, M. | 2008 | Follicular dendritic cells confirm lymphoid organization in the minor salivary glands of primary Sjogren’s syndrome. | out of interest |
| Liu, J. | 2015 | The expression of death decoy receptor 3 was increased in the patients with primary Sjögren’s syndrome. | out of interest |
| Matsumura, R. | 2001 | Glandular and extraglandular expression of costimulatory molecules in patients with Sjögren’s syndrome. | out of interest |
| Perez, P. | 2000 | Differential expression of matrix metalloproteinases in labial salivary glands of patients with primary Sjögren’s syndrome. | out of interest |
| Riccieri, V. | 2001 | Immunohistologic markers of immune activation and changes of glycosylation of serum proteins in primary Sjögren's syndrome | out of interest |
| Salomonsson, S. | 2009 | Minor salivary gland immunohistology in the diagnosis of primary Sjogren’s syndrome. | out of interest |
| Szabo, K. | 2014 | The Histopathology of Labial Salivary Glands in Primary Sjögren’s Syndrome: Focusing on Follicular Helper T Cells in the Inflammatory Infiltrates | out of interest |
| Teppo, H. | 2007 | A follow-up study of minimally invasive lip biopsy in the diagnosis of Sjögren’s syndrome. | out of interest |
| Wicheta, S. | 2017 | Minor salivary gland biopsy—an important contributor to the diagnosis of Sjogren’s syndrome. | out of interest |
| Wicheta, S. | 2019 | Discrepancies in Interpretation of the Minor Salivary Gland Biopsy in the Diagnosis of Sjogren Syndrome. | out of interest |
| Zheng, L. | 2009 | Association between IFN- and primary Sjogren’s syndrome. | out of interest |
| Zandbelt, M. | 2002 | The synergistic value of focus score and IgA% score of sublabial salivary gland biopsy for the accuracy of the diagnosis of Sjӧgren’s syndrome: a10-year comparison. | out of interest |
| Alunno, A. | 2014 | CD4-CD8- T-cells in primary Sjögren’s syndrome: Association with the extent of glandular involvement. | out of interest |
| Bikker, A. | 2010 | Increased Expression of Interleukin-7 in Labial Salivary Glands of Patients With Primary Sjo¨gren’s Syndrome Correlates With Increased Inflammation. | out of interest |
| Cortés, J. | 2019 | Synaptotagmin-1 overexpression under inflammatory conditions affects secretion in salivary glands from Sjögren's syndrome patients. | out of interest |
| Costa, S. | 2016 | B-cell and T-cell quantification in minor salivary glands in primary Sjögren’s syndrome: development and validation of a pixel-based digital procedure. | out of interest |
| Delli, K. | 2016 | Towards personalised treatment in primary Sjögren’s syndrome: baseline parotid histopathology predicts responsiveness to rituximab treatment. | out of interest |
| Donati, V. | 2020 | Total area of inflammatory infiltrate and percentage of inflammatory infiltrate identify different clinical-serological subsets of primary Sjögren’s syndrome better than traditional histopathological parameters. | out of interest |
| Giovelli, R. | 2015 | Clinical characteristics and biopsy accuracy in suspected cases of Sjögren’s syndrome referred to labial salivary gland biopsy. | out of interest |
| Katayama, I. | 1991 | Clinical and Histological Analysis of Labial Lip Biopsy in Sjogren Syndrome. | out of interest |
| Leehan, K. | 2018 | Minor salivary gland fibrosis in Sjögren’s syndrome is elevated, associated with focus score and not solely a consequence of aging. | out of interest |
| Liu, C. | 2017 | Characteristics of primary Sjögren’s syndrome patients with IgG4 positive plasma cells infiltration in the labial salivary glands. | out of interest |
| Nakamura, S. | 1997 | An association between salivary gland disease and serological abnormalities in Sjogren's syndrome. | out of interest |
| Seror, R. | 2015 | Low numbers of blood and salivary natural killer cells are associated with a better response to belimumab in primary Sjögren’s syndrome: results of the BELISS study. | out of interest |
| Turkcapar, N. | 2015 | Vasculitis and Expression of Vascular Cell Adhesion Molecule-1, Intercellular Adhesion Molecule-1, and E-Selectin in Salivary Glands of Patients with Sjögren’s Syndrome. | out of interest |
| Yoshimura, S. | 2015 | Abnormal distribution of AQP5 in labial salivary glands is associated with poor saliva secretion in patients with Sjögren's syndrome including neuromyelitis optica complicated patients. | out of interest |
| Zandebelt, M. | 2002 | Abnormal distribution of AQP5 in labial salivary glands is associated with poor saliva secretion in patients with Sjögren's syndrome including neuromyelitis optica complicated patients. | out of interest |
| Chen, W. | 2015 | Local and Systemic IKK𝜀 and NF-𝜅B Signaling Associated with Sjögren’s Syndrome Immunopathogenesis. | out of interest |
| Christodoulou, M. | 2008 | Foxp3T-Regulatory Cells in Sjo¨gren’s Syndrome. | out of interest |
| Christodoulou, M. | 2010 | Characteristics of the minor salivary gland infiltrates in Sjogren's syndrome. | out of interest |
